# Robust ΦC31-Mediated Genome Engineering in *Drosophila melanogaster* Using Minimal attP/attB Phage Sites

**DOI:** 10.1534/g3.118.200051

**Published:** 2018-03-09

**Authors:** Roumen Voutev, Richard S. Mann

**Affiliations:** Departments of Biochemistry and Molecular Biophysics and Systems Biology, Jerome L. Greene Science Center, Columbia University, New York, NY 10027

**Keywords:** ΦC31 recombinase, cassette exchange, CRISPR/Cas9, genome editing, *D. melanogaster*, attB/attP sites, Genome Report

## Abstract

Effective genome engineering should lead to a desired locus change with minimal adverse impact to the genome itself. However, flanking loci with site-directed recombinase recognition sites, such as those of the phage ΦC31 integrase, allows for creation of platforms for cassette exchange and manipulation of genomic regions in an iterative manner, once specific loci have been targeted. Here we show that a genomic locus engineered with inverted minimal phage ΦC31 attP/attB sites can undergo efficient recombinase-mediated cassette exchange (RMCE) in the fruit fly *Drosophila melanogaster*.

The introduction of CRISPR/Cas9 genome editing technique as an everyday molecular biology tool has opened enormous future opportunities for both biological research and gene therapy (reviewed in delker and mann 2017). As a supplement to this tool, it could be very advantageous to be able to reiteratively modify a locus of interest once it has already been targeted with the CRISPR/Cas9 system. One way to achieve such versatility is by flanking the targeted locus with phage attP or attB sites of one of the already extensively researched site-directed recombinases such as ΦC31 (groth *et al.* 2000) or Bxb1 (ghosh *et al.* 2003; kim *et al.* 2003). Subsequently, the resulting attP(attB)-flanked allele could be edited with admirable precision through recombinase-mediated cassette exchange (RMCE) without adverse effects to the genome, as long as the attP/attB scars do not cause significant DNA/chromatin changes.

## Methods & Materials

*Drosophila melanogaster* strain *M[vas-int.Dm]ZH-2A* (#40161, Bloomington Drosophila Stock Center, Bloomington, IN) was used as a source of germline integrase.

**Figure 1:**
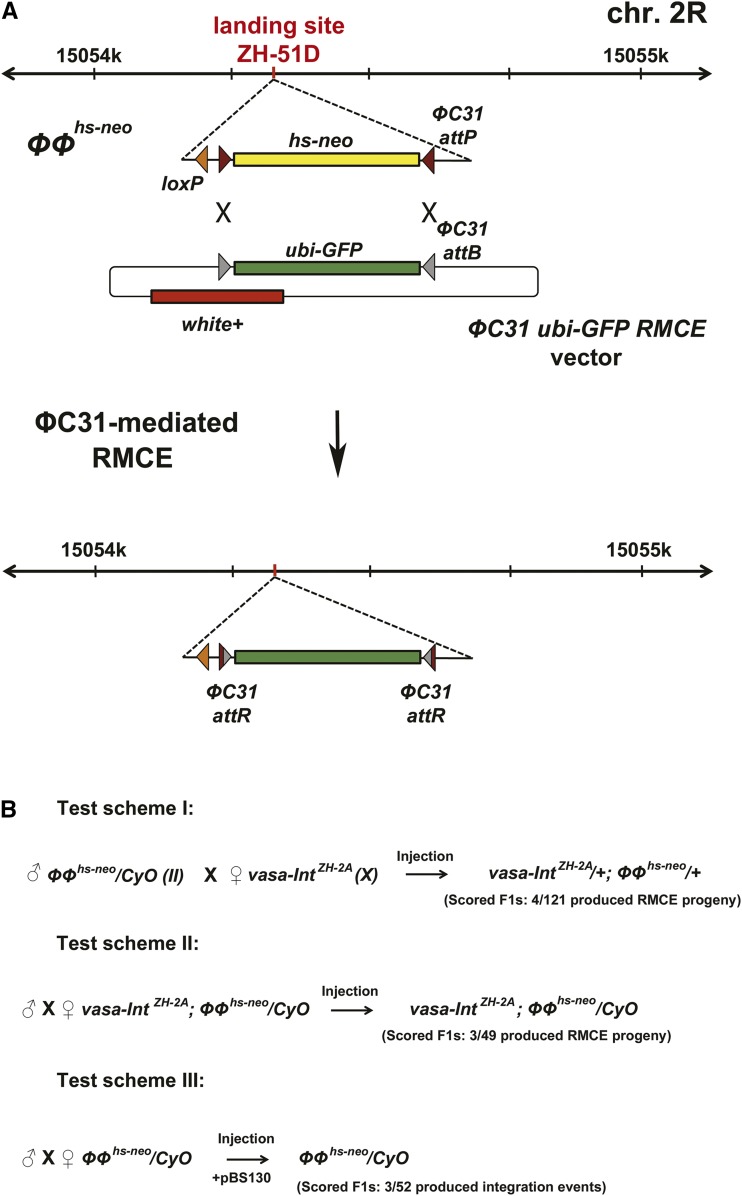
Genome engineering by using minimal ΦC31 attP/attB sites. A) Schematic representation of the ZH-51D landing site locus on chromosome 2R and the genome engineering of the *ΦΦ^hs-neo^* allele using ΦC31-catalyzed recombinase-mediated cassette exchange (RMCE). Brown represents a 39bp ΦC31 attP site; gray represents a 36bp ΦC31 attB site. B) Injection schemes employed in this study. Homozygous *ΦΦ^hs-neo^* F1 fertile animals segregating in test schemes II and III were included in the scoring as well.

39bp ΦC31 attP site (CCCCAACTGGGGTAACCTTTGAGTTCTCTCAGTTGGGGG) was introduced in vector pRVV598 (#87629, www.addgene.org; (voutev and mann 2017) in forward and reverse orientation (Figure 1A), respectively, flanking a *hs-neo* cassette and replacing the Bxb1 attP sites in vector pRVV598. A loxP site was introduced ahead of this cassette and the resulting vector was used for injection and creation of the allele *ΦΦ^hs-neo^*. The *ΦC31 ubi-GFP RMCE* vector (Figure 1A) was created by replacing the Bxb1 attP sites in vector pRVV651 (#87631, www.addgene.org; (voutev and mann 2017)) with 36bp ΦC31 attB sites (GGGTGCCAGGGCGTGCCCTTGGGCTCCCCGGGCGCG) in forward and reverse orientation, respectively, thus flanking a *Ubi-GFP* cassette (Figure 1A). Plasmid DNA, maps, and complete vector sequences are made available at Addgene (Cambridge, MA, USA; www.addgene.org); Addgene vector IDs: 108279, 108280, 108281, 108282, and 108283.

### Data availability

The authors state that all data necessary for confirming the conclusions presented in the article are represented fully within the article.

## Results and Discussion

The ΦC31 site-directed recombinase has already become a common tool in fly genetics for both genome plasmid integration (groth *et al.* 2004) and RMCE (venken *et al.* 2011). In addition, 54 bp attB and 50 bp attP ΦC31 sites have been demonstrated to mediate efficient plasmid integration events in *D. melanogaster* (huang *et al.* 2009) but these sites have not been tested for RMCE. Moreover, 40 bp attB/220 bp attP pairs of ΦC31 sites are capable of performing efficient RMCE in the fruit fly (bateman and wu 2008). However, even shorter 34 bp attB and 39 bp attP ΦC31 sites have been shown to function in *E. coli* with close to 100% efficiency, while lowering further the number of base pairs of these sites reduces the efficiency of recombination dramatically (groth *et al.* 2000). Here we test if shorter ΦC31 att sites would function in *D. melanogaster* for RMCE, because such sites would in principle diminish the effects of the exogenous DNA to a locus of interest during genome engineering.

The features of each locus should carefully be considered before introducing any exogenous attP/attB sites. For example, an enhancer element of interest that is controlled by Mad/Smad (Mothers against Dpp) proteins would not be feasible for flanking and further study with the 54 bp ΦC31 attB site (huang *et al.* 2009) because it contains a consensus GCCGCGGT Mad binding site (kim *et al.* 1997). In addition, this attB site contains a putative splice donor (agccgcgGTGCGGGT, in vector pGE-attB (huang *et al.* 2009)) with a 0.29 score (score ranges from 0 to 1, (reese *et al.* 1997)), which might interfere with splicing if introduced as a flank within introns/exons or eRNAs. Using longer attB and attP sites exponentially increases the number of putative transcription factor (TF) binding sites or other regulatory sites, which prevents them from being a viable option for flanking certain loci. For example, the commonly used in RMCE 101 bp attB sites (venken *et al.* 2011) contain additional putative splice donor sites in both the forward and reverse DNA strand (tagcgatGTAGGTCA (0.56 score) and cagatggGTGAGGTG (0.70 score (reese *et al.* 1997)) respectively, in vector pBS-KS-attB1-2 (venken *et al.* 2011)) and many more putative transcription factor sites from diverse TF families (FlyFactorSurvey, (zhu *et al.* 2011)). Thus, we decided to test minimal attB/attP sites for RMCE and creation of platforms for cassette exchange in order to strongly reduce the number of transcription factors and other DNA/RNA-binding regulatory proteins that could potentially bind to these exogenous sequences.

To test minimal ΦC31 sites in RMCE, we used as a starting point the *BB^hs-neo^* allele that we previously created (voutev and mann 2017) in landing site ZH-51D (bischof *et al.* 2007). We introduced through Bxb1-mediated integration in the distal (right) Bxb1 attP site (voutev and mann 2017) a plasmid containing the selectable marker *hs-neo* (steller and pirrotta 1985) flanked by inverted 39bp ΦC31 attP sites (Figure 1A). We also positioned a loxP site ahead of this cassette (Figure 1A) that allowed us to excise all intervening plasmid DNA (and the leftover cassette from *BB^hs-neo^*) through Cre/loxP-mediated excision, which is characteristic for landing site ZH-51D (bischof *et al.* 2007). Thus, we converted the *BB^hs-neo^* into a clean allele of *hs-neo* flanked by minimal inverted ΦC31 attP sites (Figure 1A), which we called *ΦΦ^hs-neo^*.

We also created a compatible ΦC31 RMCE vector that contains *ubiquitin-GFP* (*ubi-GFP*) cassette flanked by inverted minimal 36 bp ΦC31 attB sites (Figure 1A). In addition, this *ΦC31 ubi-GFP RMCE* vector contains *white* (*w+*) selectable marker (Figure 1A) that allows for visually differentiating between vector integration events and RMCE events.

Next, we tested the RMCE efficiency between the *ΦC31 ubi-GFP RMCE* vector (injected at 250 ng/μl) and the *ΦΦ^hs-neo^* allele in fruit fly embryos by providing germline expression of the ΦC31 recombinase in three different ways (Figure 1B). First, we crossed *ΦΦ^hs-neo^/CyO* males to *M[vas-int.Dm]ZH-2A (X)* females (bischof *et al.* 2007) and injected 400 of the resulting embryos from this cross. Second, we established a *M[vas-int.Dm]ZH-2A*; *ΦΦ^hs-neo^/CyO* strain and injected 200 embryos laid by these flies. Third, we co-injected the *ΦC31 ubi-GFP RMCE* vector together with the pBS130 plasmid (a source of germline ΦC31 integrase (gohl *et al.* 2011)) at 250:100 ng/μl ratio into 200 embryos laid by the *ΦΦ^hs-neo^/CyO* strain. We raised the larvae resulting from each injection at 25° and crossed each hatched individual to *yw* flies (we crossed only the non-*CyO* flies hatching from the first injection scenario).

We scored the progeny of each injected fertile individual for successful RMCE events by the ubiquitous expression of GFP from the *ubi-GFP* cassette. Simultaneously, we could detect any integration *vs.* RMCE events through the presence of the *w+* marker in the fly eyes. In the first case, where each individual was a result of the cross between *ΦΦ^hs-neo^/CyO* males and *M[vas-int.Dm]ZH-2A* females (Figure 1B), we detected 3.3% RMCE events (4/121 individuals) and each RMCE positive parent was segregating equally complete RMCE and integration events. We sequence-verified four RMCE fly lines and the *ubi-GFP* cassette was exchanged in both forward and reverse orientation, as expected.

In the second case, where we injected *ΦC31 ubi-GFP RMCE* vector into the *M[vas-int.Dm]ZH-2A*; *ΦΦ^hs-neo^/CyO* established strain (Figure 1B) we detected higher percentage of RMCE events: 6.1% (3/49 individuals). In addition, only one individual was segregating both RMCE and integration events while the other two individuals were segregating only RMCE events.

Interestingly, in the case where the source of integrase was provided through a co-injected vector (pBS130) rather than an established stock (Figure 1B), we detected only integration events, 5.8% (3/52 individuals), and no full RMCE events. However, we found that each integration allele could be lead to a complete RMCE event through intra-molecular recombination between the intact ΦC31 attP/attB sites left at the locus. This can occur by introducing/maintaining the integrated allele in the background of the *M[vas-int.Dm]ZH-2A* source of integrase. Surprisingly, such events occurred at much lower rate for ΦC31 (2/100 progeny) than in the case of Bxb1 recombinase (67/100 progeny (voutev and mann 2017)), which might be due to differences in the recombination mechanism between the two recombinase systems (thorpe and smith 1998; ghosh *et al.* 2003). This property of the ΦC31 recombinase might be useful in experiments where a low-rate switch between an integration allele and an RMCE allele is desired.

Taken together, our results show that using minimal attP/attB ΦC31 for RMCE is feasible in *D. melanogaster*. Although the rate of RMCE decreases around ten-fold in comparison with the RMCE rates when using longer ΦC31 sites (Venken *et al.* 2011), injecting only 200 embryos is sufficient to generate multiple RMCE fly lines and has the advantage of not introducing unnecessary sequences that might interfere with gene/locus function of the engineered allele. Furthermore, in genome editing it is always better to introduce minimal amount of exogenous DNA since other unforeseeable chromatin disruptions may occur. The orientation of the introduced attB/attP sites should also be taken into account in genome engineering: for example, the core of the attP site contains a consensus Trithorax-like (Trl) binding site, GTTCTCTCAG (zhu *et al.* 2011), which could potentially lead to binding of Trx group proteins and consequent chromatin remodeling of a locus of interest. However, if the attB/attP ΦC31 sites are oriented in the manner shown in Figure 1A, this sequence would be eliminated during the recombination reaction and conversion to an attR site (Figure 1A).

Our findings are applicable to many other organisms as the ΦC31 recombinase is being widely used and similar considerations over flanking of loci with attB/attP sites are highly relevant in other biological contexts. Analogous analysis of other recombinase systems and sites is recommended in each particular genomic locus engineering case when exogenous sites are being used.

## 
